# Alcohol consumption and the risk of Barrett’s esophagus: a comprehensive meta-analysis

**DOI:** 10.1038/srep16048

**Published:** 2015-11-06

**Authors:** Lin-Lin Ren, Ting-Ting Yan, Zhen-Hua Wang, Zhao-Lian Bian, Fan Yang, Jie Hong, Hao-Yan Chen, Jing-Yuan Fang

**Affiliations:** 1 State Key Laboratory of Oncogenes and Related Genes, Division of Gastroenterology and Hepatology, Ren Ji Hospital, School of Medicine, Shanghai Jiao Tong University, Shanghai Institute of Digestive Disease. 145 Middle Shandong Rd, Shanghai, 200001, China

## Abstract

Several studies have been proposed to investigate the association between alcohol consumption and risk of Barrett’s esophagus (BE), but as of yet, no quantitative summary of the literature to clarify the relationship between them. In our study, twenty eligible cohort studies involving 42925 participants were identified. Combined relative risk (RR) ratios for the highest versus lowest alcohol consumption levels were calculated. The alcohol dose-response analysis was performed to investigate the association between the increment consumption of 10 g/d alcohol and the risk of developing BE. Subgroup analyses were used to examine heterogeneity across the studies. A combined RR of 0.98 (0.62–1.34) was found when comparing highest vs. lowest alcohol consumption levels for BE. An inverse association between alcohol and incidence of BE (RR 0.51; 95% CI: 0.055–0.96) was demonstrated in women. Moreover, Asian drinkers had a relative higher risk of BE (RR 1.34; 95% CI: 1.11–1.56) compared with Western drinkers. In conclusion, our results showed that overall alcohol consumption was not associated with increased BE incidence. The limited data available on alcohol consumption supports a tentative inversion of alcohol consumption with BE risk in women, while Asian drinkers tend to have a higher risk of BE.

Barrett’s esophagus (BE) is defined as a change in the distal esophageal epithelium of any length characterized by columnar type mucosa during endoscopy and is further confirmed to have intestinal metaplasia by histopathological examinations^1^. BE is considered as a complication of chronic GERD (gastroesophageal reflux disease) and to predispose to EAC (esophageal adenocarcinoma)^2^, therefore, it is of high clinical significance to investigate the risk factors for BE.

BE, affects approximately 2% of the Western population, is most prevalent disease in white men aged over 50. The incidence of BE has progressively increased in recent decades, especially in Western countries. The reasons for this are largely unknown, and several lifestyle and dietary risk factors, including BMI (body mass index)^3^,^4^, alcohol intake^5^,^6^, smoking^6^,^7^, job type, education^8^ and drug use, have been proposed and investigated thoroughly. Potentially, modifiable risk factors for BE include gastroesophageal reflux disease and abdominal obesity, while use of aspirin and non-steroidal anti-inflammatory drugs (NSAIDs) may prevent the development of BE.

Among these lifestyle factors, the association between alcohol and risk of BE remains unclear and even controversial^9^. Higher alcohol consumption was once reported to be positively associated with BE^10^,^11^,^12^. By contrast, data from other studies had generally reported no association between alcohol consumption and risk of BE^3^,^4^,^8^; Moreover, results among studies reporting beverage-specific effects on BE were conflicting. Some have reported that BE was inversely associated with wine consumption^4^,^8^, while others have found that people who consumed liquor would have a high risk of BE^4^.

In light of the inconsistent findings to date, there is a need to comprehensively investigate the associations between alcohol and BE, as well as the quantification of alcohol effects on BE. To this end, we performed a comprehensive meta-analysis of previous studies to evaluate the possible relationship between alcohol consumption and the risk of BE.

## Results

### Search results and characteristics of included studies

**Figure 1:**
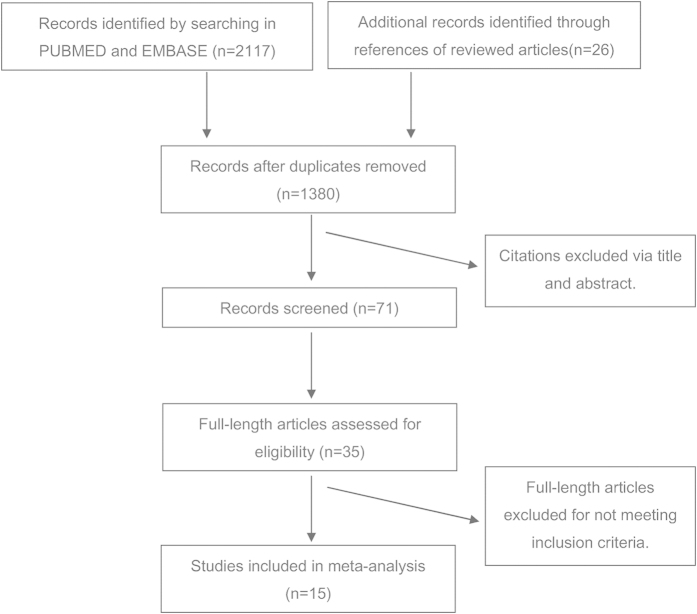
The selection flowchart of relevant studies for meta-analysis.

From 2117 studies initially identified, 15 articles met our inclusion criteria (Fig. 1). Altogether twenty studies involving 42925 participants and 3775 cases of BE were identified according to the inclusion criteria in the pooled-analysis. The characteristics of these studies are summarized in Supplementary Table 1.

A total of 41593 subjects were from Western countries^3^,^4^,^5^,^6^,^7^,^8^,^9^,^10^,^11^,^12^,^13^,^14^,^15^ and 1332 subjects^16^,^17^ were from Asia. Among them, six studies provided RRs for BE in men (7593 cases)^3^,^5^,^7^,^15^,^17^ and five studies provided RRs for women (7130 cases)^3^,^5^,^7^,^15^, respectively. Four different types of controls were used in these studies. Among them, Population based control (30176 cases)^3^,^4^,^9^,^12^ used in five studies, endoscopic negative control (3683 cases)^6^,^11^,^14^,^17^ in five studies , mixed control (5719 cases)^10^,^13^,^16^ in three studies, and inflammation control (3347 cases)^5^,^7^,^8^,^15^ in eight studies.

### The BE incidence in highest level compared with lowest level of alcohol consumption

**Figure 2:**
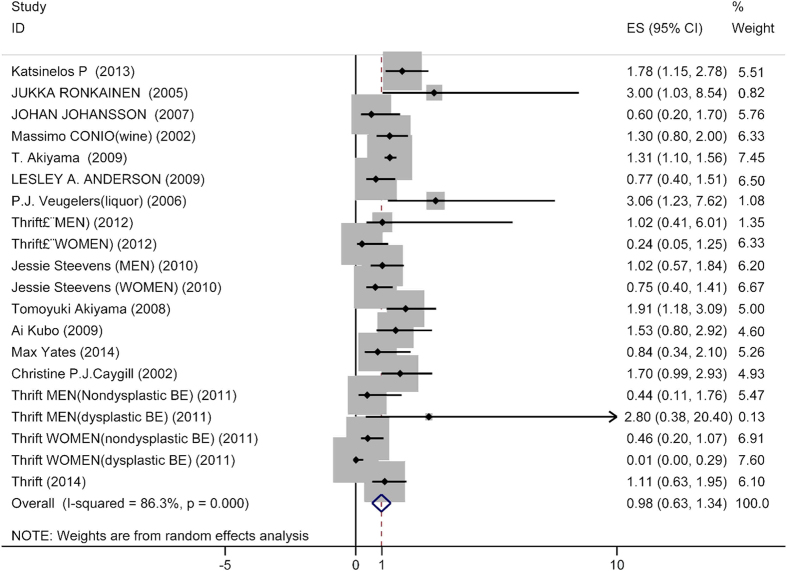
Forest plot of studies of the risk of BE for highest vs. lowest alcohol consumption. The size of the data markers (squares) corresponds to the weight of the study in the meta-analysis. The combined relative risk is calculated using the random effects method.

The combined RR of 0.98 (95% CI: 0.62–1.34) was determined from twenty studies comparing highest versus lowest alcohol consumption levels against cases of BE (Fig. 2). Stratified results are shown in Table 1 by geographic region, sex and control types. When subgrouping by geographic region, a positive association between higher alcohol consumption and incidence of BE (RR: 1.34; 95% CI: 1.11–1.56) was demonstrated in the Asian studies. However, no significant association was revealed in the population from western countries.

Interestingly, women who consumed more alcohol were less likely to suffer from BE (RR: 0.51; 95% CI: 0.055–0.96). As per the studies in men, no statistically significant findings could be noted (RR: 1.17; 95% CI: 0.82–1.53).

We further subgrouped the subjects based on the type of control used in these studies, and found no significant tendency of higher alcohol consumption to higher risk of BE in population control (RR: 0.848; 95% CI: 0.58–1.15), inflammation control (RR: 0.58; 95% CI: 0.13–1.02), mixed control (RR: 1.38; 95% CI: 0.99–1.77), or negative control (RR: 1.18; 95% CI: 0.84–1.53), respectively.

### Increment of 10 g/d alcohol consumption with BE incidence

**Figure 3:**
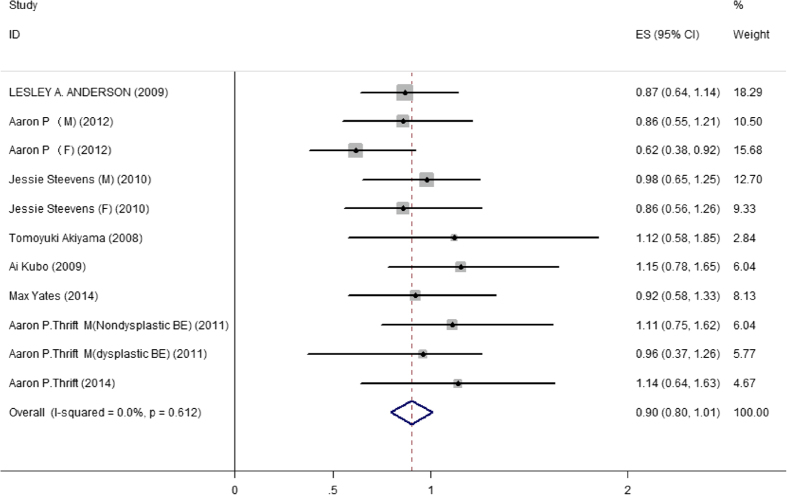
Forest plot of studies of the risk of BE increment of 10 g/d for alcohol consumption. The size of the data markers (squares) corresponds to the weight of the study in the meta-analysis. The combined relative risk is calculated using the random effects method.

By using the dose-response analysis, we did not find the association between an increase in alcohol consumption of 10 g/d and the risk of developing BE (RR: 0.90; 95% CI: 0.79–1.01) (Fig. 3). The results for the stratified analysis of the increment of 10 g/d alcohol consumption based on the geographic region are shown in Table 2. However, a statistically significant inversion between an increase in alcohol consumption of 10 g/d and risk of BE was indicated from studies in women (RR: 0.71; 95%  CI: 0.50–0.92).

### Publication bias

**Figure 4:**
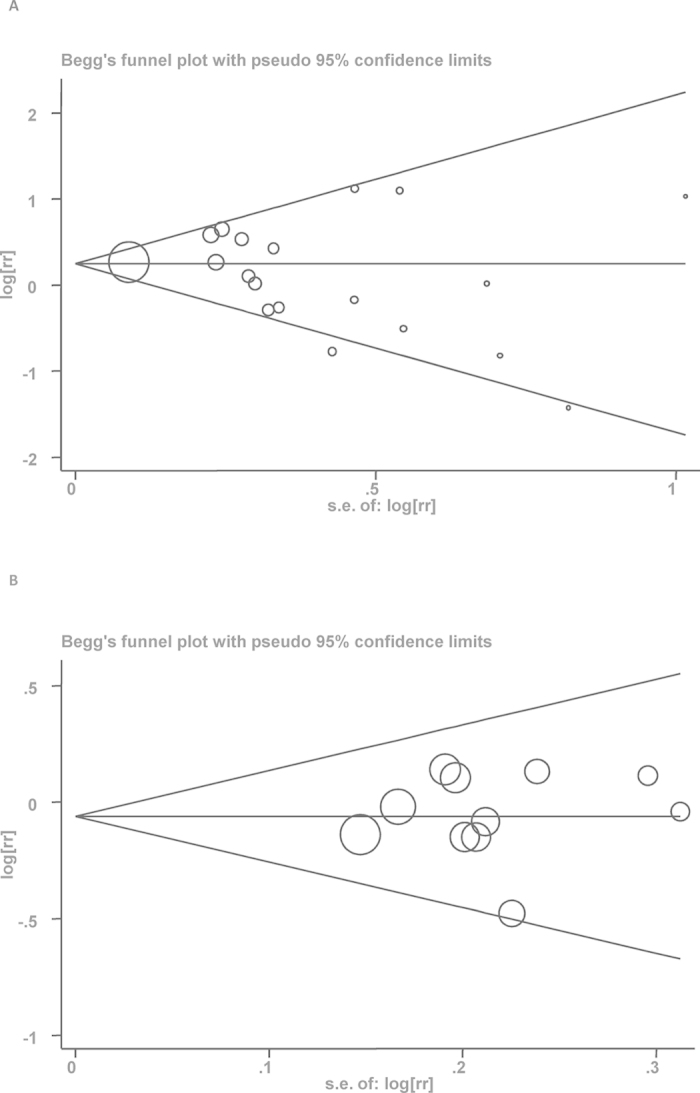
(**A**) Begg’s funnel plot of studies on highest vs. lowest alcohol consumption and BE risk. The solid line in the center is the natural logarithm of pooled relative risk ratio (RR), and two oblique lines are pseudo 95% confidence limits. SE, standard error. (**B**) Begg’s funnel plot of studies on increment of 10 g/d alcohol consumption and BE risk. The solid line in the center is the natural logarithm of pooled relative risk ratio (RR), and two oblique lines are pseudo 95% confidence limits. SE, standard error.

Publication bias was estimated by Begg’s and Egger’s tests (Table 1). There was no statistically significant publication bias in the literature on highest versus lowest level of alcohol consumption and BE (Fig. 4A) in both sexes, as well as increment of 10 g/d alcohol consumption and incidence of BE (Fig. 4B).

## Discussion

In the past century, numerous publications have demonstrated the association between alcohol consumption and the incidence of BE; however, the results are largely controversial^6^,^17^. As far as we know, this is the first pooled study to provide comprehensive evidence of the association between alcohol consumption and BE incidence. Our analyses included the combined data from twenty studies reporting 3775 cases of BE, which offered more precise risk estimates. Our results showed that overall alcohol consumption was not associated with increased BE incidence after pooling all data from highest versus lowest comparisons, which is in line with several previous studies^3^,^14^,^18^.

Subgroup analyses showed that Asians who consumed higher level of alcohol had a relative higher incidence of BE than those who never drank alcohol. However, the same tendency was not observed in Western countries. The similar result was demonstrated in the dose-response analysis, including highest vs. lowest analysis and subgroup analysis. A major explanation of this phenomenon is the disparity in alcohol sensitivity among different ethnic groups. Ethanol is metabolized to acetaldehyde within esophageal mucosa, which is determined by the alcohol dehydrogenase^19^. Previous studies revealed that the distribution of human liver alcohol dehydrogenase (ADH2) and the aldehyde dehydrogenase (ALDH2) differs in different populations^20^. The polymorphism of the ALDH2 gene plays a central role in Asian alcohol hypersensitivity and has been associated with the risk for esophageal cancer. The inactive form of aldehyde dehydrogenase-2 (ALDH2), encoded by the gene ALDH2*1/2*2, which is prevalent in Asians, exposes Asians to higher levels of acetaldehyde, an established animal carcinogen, and was a strong risk factor for both synchronous and metachronous multiple esophageal cancers among Asia drinkers. Moreover, the polymorphism of alcohol dehydrogenase 3(ALDH3) in Asians also plays a part in esophageal adenocarcinoma^18^. As a precancerous disease of esophageal cancer, BE patients in Asian countries possessing a different phenotype of ALDH2 and ALDH3 and alcohol metabolism process may be more susceptible to alcohol than Western populations.

An inverse association between highest versus lowest level of alcohol consumption and risk of BE in women was also found in these analyses. However, no significant association was found between alcohol consumption and risk of BE in men. Recent meta-analyses confirmed the existence of a strong relationship between alcohol and hormone-related diseases including cancers^2^,^21^,^22^; therefore, a role of alcohol on hormone metabolism and serum levels is plausible. Alcohol can interact with the endocrine system, and affect the homeostasis of estrogen. It has been hypothesized that estrogens play a protective role against esophageal adenocarcinoma^23^,^24^. This statement has gained further support from clinical studies^23^,^25^ and studies in mice model^26^. As a precancerous disease of esophageal adenocarcinoma, BE is also highly affected by estrogens. Experiments in esophageal adenocarcinoma and Barrett’s esophagus cells revealed that cells treated with selective estrogen receptor ligands showed decreased cell growth and increased cell apoptosis^23^. Therefore, estrogen might protect against esophageal adenocarcinoma and BE via modulating cell growth and apoptosis. Besides, alcohol metabolism may also contribute to increased production of a form of estrogen called estradiol in women rather than in men^27^. Moreover, 2-methoxyestradiol (2-ME2), an endogenous byproduct of 17β-estradiol (E2), could markedly reduce the Barrett’s esophageal adenocarcinoma (BEAC) cell proliferation by regulating apoptotic machinery such as Bcl-2 and Bax. 2-ME2 has been shown to modulate BEAC cell growth and behavior, which appears to play a critical role in the induction of antitumor responses in cancer cells^28^. Above all, alcohol consumption may lead to interruption of estrogen metabolism in liver and increase the serum level of estrogen in women^27^, which could protect women from suffering from BE via an estrogen-induced mechanism. Given the fact that men had a relative lower level of estrogen, the consumption of alcohol may have little effect on the estrogen metabolism in men. And this difference may partially account for the relative inverse relationship between alcohol consumption and BE in women instead of men. However, due to a limited number of included studies in women/men, the conclusion may be not strong enough, and more follow up studies in women are needed to clarify this phenomenon in the future.

Traditionally, GERD is divided into three types: NERD (Non-erosive Reflux Disease), RE(Reflux Esophagitis) and BE^29^. And NERD usually progresses into EC via RE or BE^12^. However, a recent hypothesis suggested that the four types of disease are very different lesions that vary from each other. Although there is already much evidence, large numbers of well-designed studies are still required for further confirmation. The subgroup data from our analyses demonstrated that the types of control used in different studies did not affect the relationship between alcohol consumption and BE, which is in consistent with the novel opinion.

Cigarette smoking and BMI are the most likely confounding factors in the relationship between alcohol consumption and BE incidence. The results of our meta-analysis, which was confined to studies that adjusted their results for potential confounding factors including smoking, education levels and body mass index, demonstrated a more precise association between alcohol consumption and BE incidence.

Several limitations should be considered in our analysis. Firstly, although we tried rescaling alcohol consumption to the number of grams daily, the methods for measuring alcohol consumption in the included trials differed across studies, resulting in attenuation of inverse association. Secondly, several attempts have been made to separate the effects of different types of alcoholic beverages. Some authors reported no apparent differences, while others reported greater risks with beer, and a lower risk with wine. Due to a limited number of such sufficiently stratified subgroup studies, we have considered only total alcohol consumption in the present overview, which could have distorted the results. Given that only few studies were included in the stratified subgroup analysis, the RRs of subgroup analysis may be partially biased to some extent and should be tested with follow up studies. Another open issue is the definition of former drinkers, which in some studies may include only a fraction of former drinkers. However, the time-risk relations between alcohol consumption and BE are complex, and misclassification of former drinkers should have led to an underestimate of the real association. It is likely, moreover, that alcohol consumption was systematically under-reported in several studies. Consequently, all the RRs may be biased towards lower levels of consumption because of selective under-reporting by cases.

Notwithstanding the limitations discussed above, this analysis still includes most of the published information on alcohol and BE, consequently providing a most accurate relative risk estimates. In summary, there is insufficient evidence from these eligible studies to conclude that alcohol consumption is significantly associated with the risk of BE. Some of the findings from this meta-analysis are innovative and of specific relevance, including an apparently stronger association of BE with alcohol consumption in Asia than in Western countries, and relative lower risk of BE for alcohol consumption in women than for alcohol consumption in men. Although some of the conclusions maybe biased, the results may also provide certain improvement of the knowledge of BE risk. Further large prospective cohort studies, with careful control of confounding factors, are needed to reach a more definitive conclusion as to whether the consumption of alcohol can lead to BE. And related researches are required to identify the underlying mechanism.

## Methods

### Search strategy

We searched the Pubmed and Embase databases from June 1966 to July 2014 for studies of alcohol consumption in relation to risk of Barrett’s esophagus that were published in English. The terms “Barrett’s Esophagus” or “Barrett Esophagus” or “Barrett’s Oesophagus” or “Barrett Oesophagus” and “alcohol” or “beverage” or “consumption” were searched as text word or MeSH terms if possible. Some studies were searched manually in the reference lists of relevant articles.

### Inclusion criteria

Studies were included if the following inclusion criteria were satisfied: 1) only studies designed as case-control, nested case-control, cohort study or cross-sectional study were included; 2) BE was inspected on endoscopy and confirmed by pathologist as specialized intestinal metaplasia (SIM); 3) RR estimates and corresponding 95%  CIs were reported in the study, or data were adequate to calculate them; 4) an inner control group was applied when calculating the risk estimate.

### Exclusion criteria.

Studies were excluded if they met any of the following criteria: 1) the full text of the article could not be obtained; 2) the necessary clinical characteristics of BE patients were insufficient, such as lack of the amount of alcohol consumption; 3) BE diagnosed only on endoscopic appearance and without histological confirmation.

### Data extraction

Data were extracted via a recognized data extraction form, collecting the following information: the first author, publication year, country, follow-up period, number of participants, sex, measurements of alcohol consumption, adjusted variables, the risk estimates and their corresponding 95%  CIs or data used to calculate them. If a study provided separate RR estimates for men and women, we treated the RRs as two different studies. If a study contained several RR estimates, we took the RR reflecting the greatest extent of control for underlying confounders. If a study applied different types of control, we choose inflammation control data preferentially, and then endoscopic negative control, mixed control; population control was the last choice. In addition, adjusted RRs were selected in preference to non-adjusted RRs. All the data were examined by two independently reviewers (Lin-Lin Ren and Ting-Ting Yan) for eligibility and quality and divergences were resolved by a third reviewer (Zhen-Hua Wang).

### Statistical analysis

Pooled RRs for highest vs. lowest categories of alcohol consumption on the risk of BE from each study were calculated by applying a random effects model. Separate RRs were calculated to compare BE cases with different control groups, namely the GERD control group, non-GERD control group, population control group and mixed control group, sex and geographic region. For dose-response analysis, a risk estimate for an increment of alcohol consumption of 10 g/day for each study was calculated to normalize the variation between studies in the difference in exposure categories using methods proposed by Greenland and Longnecker^30^. In different studies, alcohol consumption was presented by grams of alcohol/wine/beer/liquor, cups of alcohol/wine/beer/liquor consumption, or duration of alcohol/wine/beer/liquor consumption; therefore, we rescaled alcohol consumption according to published articles^31^,^32^. If a study showed the exact gram of alcohol consumption or conversion method, we transferred the alcohol consumption as described. If a study did not provide the gram of alcohol, total amount of alcohol consumed was estimated at 12.8 g for a glass, bottle, or can of beer (12 oz),11g for a glass of wine (4 oz), and 14 g for a shot of liquuor (1.5 oz). One drink was defined as 12.8 g of alcohol (median amount of alcohol in beer, wine, and liquor), as shown in Supplementary Table 1. Then we obtained the general RR estimates by pooling the separate RR using the inverse of the corresponding variance coefficient as weights.

The degree of heterogeneity across the studies was calculated via the Q statistic^33^, whose statistical significance was set at 0.10^34^. If the significant heterogeneity was less than 0.10, the random model rather than the fixed-effects model was used for further analysis. Subgroup analyses were explored to identify the causes of heterogeneity. For the publication bias, we checked the funnel plots with Begg’s test^34^ and Egger’s test^35^, and the results were regarded as statistically significant if the P value was less than 0.10^35^. All analyses were performed with Stata version 11.0 software.

## Additional Information

**How to cite this article**: Ren, L.-L. *et al.* Alcohol consumption and the risk of Barrett's esophagus: a comprehensive meta-analysis. *Sci. Rep.* **5**, 16048; doi: 10.1038/srep16048 (2015).

## Supplementary Material

Supplementary Information

## Figures and Tables

**Table 1:** Risk estimates of alcohol consumption with BE by geographic region, gender and control types.

		No. of studies	No. of total cases	No. of BE patients	Relative risk (95% CI)	Heterogeneity		Publication bias	
						P	I^2^	Begg’s test	Egger’s test
	Total of Barrett esophagus	20	42925	3775	0.98 (0.62–1.34)	0.00	86.3%	0.16	0.28
Geographic region	Asian	2	1332	585	1.34 (1.11–1.56)	0.22	32.0%	1.00	—
	Western countries	18	41593	3190	0.88 (0.54–1.22)	0.00	77.6%	0.23	0.23
Gender	MEN	6	7593	1250	1.17(0.82–1.53)	0.28	20.8%	0.71	0.48
	WOMEN	5	7130	746	0.51 (0.06–0.96)	0.00	80.8%	0.31	0.19
Control types	Population control	5	30176	1328	0.85 (0.55–1.15)	0.67	0.00%	0.46	0.33
	Inflammation control	8	3347	1343	0.58 (0.13–1.02)	0.002	69.5%	0.37	0.25
	Mixed control	3	5719	465	1.38 (0.99–1.77)	0.36	3.2%	0.30	0.34
	Endoscopic negative control	4	3683	639	1.18 (0.84–1.53)	0.19	37.5%	0.31	0.32

**Table 2:** Risk estimates of alcohol consumption with BE by geographic region, gender and control types.

	No. of studies	No. of cases	Relative risk (95% CI)	Heterogeneity		Publication bias	
				p	I^2^	Begg’s test	Egger’s test
Western countries	17	40993	0.90 (0.79–1.01)	0.56	0.00%	1.00	0.99
Asia countries	1	463	1.12 (0.49–1.85)	NA	NA	NA	NA
MEN	6	7593	0.97 (0.80–1.14)	0.90	0.00%	0.81	0.77
WOMEN	5	7130	0.71 (0.50–0.92)	0.29	11.7%	1.00	NA
Population control	5	30176	0.90 (0.73–1.07)	0.83	0.0%	1.00	0.95
Inflammation control	8	3347	0.86 (0.70–1.02)	0.18	35.5%	0.22	0.63
Endoscopic negative control	4	3683	1.13 (0.74–1.52)	0.96	0.00%	1.00	NA

NA, not applicable.

## References

[b1] Sampliner R. E. & Practice Parameters Committee of the American College of, G. Updated guidelines for the diagnosis, surveillance, and therapy of Barrett’s esophagus. The American journal of gastroenterology 97, 1888–1895, doi: 10.1111/j.1572-0241.2002.05910.x (2002). 12190150

[b2] Friedenreich C. M. *et al.* Case-control study of lifetime alcohol consumption and endometrial cancer risk. Cancer causes & control: CCC 24, 1995–2003, doi: 10.1007/s10552-013-0275-0 (2013). 23929278 PMC3824213

[b3] Steevens J. *et al.* A prospective cohort study on overweight, smoking, alcohol consumption, and risk of Barrett’s esophagus. Cancer epidemiology, biomarkers & prevention: a publication of the American Association for Cancer Research, cosponsored by the American Society of Preventive Oncology 20, 345–358, doi: 10.1158/1055-9965.EPI-10-0636 (2011). 21173169

[b4] Yates M. *et al.* Body mass index, smoking, and alcohol and risks of Barrett’s esophagus and esophageal adenocarcinoma: a UK prospective cohort study. Digestive diseases and sciences 59, 1552–1559, doi: 10.1007/s10620-013-3024-z (2014). 24500448 PMC4067535

[b5] Thrift A. P. *et al.* Lifetime alcohol consumption and risk of Barrett’s Esophagus. The American journal of gastroenterology 106, 1220–1230, doi: 10.1038/ajg.2011.89 (2011). 21427711

[b6] Thrift A. P., Kramer J. R., Richardson P. A. & El-Serag H. B. No significant effects of smoking or alcohol consumption on risk of Barrett’s esophagus. Digestive diseases and sciences 59, 108–116, doi: 10.1007/s10620-013-2892-6 (2014). 24114046 PMC3976430

[b7] Caygill C. P. *et al.* Lifestyle factors and Barrett’s esophagus. The American journal of gastroenterology 97, 1328–1331, doi: 10.1111/j.1572-0241.2002.05768.x (2002). 12094845

[b8] Kubo A. *et al.* Alcohol types and sociodemographic characteristics as risk factors for Barrett’s esophagus. Gastroenterology 136, 806–815, doi: 10.1053/j.gastro.2008.11.042 (2009). 19111726 PMC2675884

[b9] Anderson L. A. *et al.* The association between alcohol and reflux esophagitis, Barrett’s esophagus, and esophageal adenocarcinoma. Gastroenterology 136, 799–805, doi: 10.1053/j.gastro.2008.12.005 (2009). 19162028

[b10] Ronkainen J. *et al.* Prevalence of Barrett’s esophagus in the general population: an endoscopic study. Gastroenterology 129, 1825–1831, doi: 10.1053/j.gastro.2005.08.053 (2005). 16344051

[b11] Conio M. *et al.* Risk factors for Barrett’s esophagus: a case-control study. International journal of cancer. Journal international du cancer 97, 225–229 (2002). 11774268 10.1002/ijc.1583

[b12] Veugelers P. J., Porter G. A., Guernsey D. L. & Casson A. G. Obesity and lifestyle risk factors for gastroesophageal reflux disease, Barrett esophagus and esophageal adenocarcinoma. Diseases of the esophagus: official journal of the International Society for Diseases of the Esophagus/I.S.D.E 19, 321–328, doi: 10.1111/j.1442-2050.2006.00602.x (2006). 16984526

[b13] Katsinelos P. *et al.* Prevalence of Barrett’s esophagus in Northern Greece: A Prospective Study (Barrett’s esophagus). Hippokratia 17, 27–33 (2013). 23935340 PMC3738273

[b14] Johansson J. *et al.* Risk factors for Barrett’s oesophagus: a population-based approach. Scandinavian journal of gastroenterology 42, 148–156, doi: 10.1080/00365520600881037 (2007). 17327933

[b15] Thrift A. P. *et al.* A clinical risk prediction model for Barrett esophagus. Cancer prevention research 5, 1115–1123, doi: 10.1158/1940-6207.CAPR-12-0010 (2012). 22787114 PMC3750988

[b16] Akiyama T. *et al.* Risk factors for the progression of endoscopic Barrett’s epithelium in Japan: a multivariate analysis based on the Prague C & M Criteria. Digestive diseases and sciences 54, 1702–1707, doi: 10.1007/s10620-008-0537-y (2009). 19003532

[b17] Akiyama T. *et al.* Alcohol consumption is associated with an increased risk of erosive esophagitis and Barrett’s epithelium in Japanese men. BMC gastroenterology 8, 58, doi: 10.1186/1471-230X-8-58 (2008). 19077221 PMC2615024

[b18] Terry M. B. *et al.* Alcohol dehydrogenase 3 and risk of esophageal and gastric adenocarcinomas. Cancer causes & control: CCC 18, 1039–1046, doi: 10.1007/s10552-007-9046-0 (2007). 17665311

[b19] Glade M. J. Food, nutrition, and the prevention of cancer: a global perspective. American Institute for Cancer Research/World Cancer Research Fund, American Institute for Cancer Research, 1997. Nutrition 15, 523–526 (1999). 10378216 10.1016/s0899-9007(99)00021-0

[b20] Goedde H. W. *et al.* Distribution of ADH2 and ALDH2 genotypes in different populations. Human genetics 88, 344–346 (1992). 1733836 10.1007/BF00197271

[b21] Park S. Y. *et al.* Alcohol consumption and breast cancer risk among women from five ethnic groups with light to moderate intakes: the Multiethnic Cohort Study. International journal of cancer. Journal international du cancer 134, 1504–1510, doi: 10.1002/ijc.28476 (2014). 24037751 PMC4102309

[b22] Liu Y. *et al.* Alcohol intake between menarche and first pregnancy: a prospective study of breast cancer risk. Journal of the National Cancer Institute 105, 1571–1578, doi: 10.1093/jnci/djt213 (2013). 23985142 PMC3797023

[b23] Sukocheva O. A., Wee C., Ansar A., Hussey D. J. & Watson D. I. Effect of estrogen on growth and apoptosis in esophageal adenocarcinoma cells. Dis Esophagus 26, 628–635, doi: 10.1111/dote.12000 (2013). 23163347

[b24] Chandanos E. & Lagergren J. The mystery of male dominance in oesophageal cancer and the potential protective role of oestrogen. Eur J Cancer 45, 3149–3155, doi: 10.1016/j.ejca.2009.09.001 (2009). 19804965

[b25] Mathieu L. N., Kanarek N. F., Tsai H. L., Rudin C. M. & Brock M. V. Age and sex differences in the incidence of esophageal adenocarcinoma: results from the Surveillance, Epidemiology, and End Results (SEER) Registry (1973-2008). Dis Esophagus, doi: 10.1111/dote.12147 (2013). PMC3979505 24118313

[b26] Kobayashi K. [Effect of sex hormone on the experimental induction of esophageal cancer]. Nihon Geka Gakkai Zasshi 86, 280–289 (1985). 3982381

[b27] Devaud L. L., Risinger F. O. & Selvage D. Impact of the hormonal milieu on the neurobiology of alcohol dependence and withdrawal. J Gen Psychol 133, 337–356, doi: 10.3200/GENP.133.4.337–356 (2006). 17128955

[b28] Kambhampati S. *et al.* 2-methoxyestradiol inhibits Barrett’s esophageal adenocarcinoma growth and differentiation through differential regulation of the beta-catenin-E-cadherin axis. Mol Cancer Ther 9, 523–534, doi: 10.1158/1535–7163.MCT-09-0845 (2010). 20197389 PMC2837538

[b29] Rohof W. O., Hirsch D. P. & Boeckxstaens G. E. Pathophysiology and management of gastroesophageal reflux disease. Minerva gastroenterologica e dietologica 55, 289–300 (2009). 19829285

[b30] Greenland S. & Longnecker M. P. Methods for trend estimation from summarized dose-response data, with applications to meta-analysis. American journal of epidemiology 135, 1301–1309 (1992). 1626547 10.1093/oxfordjournals.aje.a116237

[b31] Qureshi A. A., Dominguez P. L., Choi H. K., Han J. & Curhan G. Alcohol intake and risk of incident psoriasis in US women: a prospective study. Archives of dermatology 146, 1364–1369, doi: 10.1001/archdermatol.2010.204 (2010). 20713772 PMC3017376

[b32] Smith-Warner S. A. *et al.* Alcohol and breast cancer in women: a pooled analysis of cohort studies. JAMA : the journal of the American Medical Association 279, 535–540 (1998). 9480365 10.1001/jama.279.7.535

[b33] DerSimonian R. & Laird N. Meta-analysis in clinical trials. Controlled clinical trials 7, 177–188 (1986). 3802833 10.1016/0197-2456(86)90046-2

[b34] Hedges L. V. & Pigott T. D. The power of statistical tests in meta-analysis. Psychological methods 6, 203–217 (2001). 11570228

[b35] Egger M., Davey Smith G., Schneider M. & Minder C. Bias in meta-analysis detected by a simple, graphical test. Bmj 315, 629–634 (1997). 9310563 10.1136/bmj.315.7109.629 PMC2127453

