# Supplementary material for: Alcohol consumption and the risk of Barrett’s esophagus: a comprehensive meta-analysis
Source: Sci Rep. 2015 Nov 6;5:16048. doi: 10.1038/srep16048 (PMC4635354; doi:10.1038/srep16048)
Supplement: Supplementary Information [file srep16048-s1.doc]

**Title page**

**Alcohol consumption and the risk of Barrett’s esophagus:** **a comprehensive meta-analysis**

Lin-Lin Ren†, Ting-Ting Yan†, Zhen-Hua Wang†, Zhao-Lian Bian, Fan Yang, Jie Hong^*^, Hao-Yan Chen^*^, Jing-Yuan Fang^*^.

Supplementary Table 1.Alcohol consumption and BE risk

| First author (year) | | | Population of Country | | | | Follow-up period | | | | | Study subjects | | | | No. of subjects in low/high level | | | Type of controls | RR (95% CI) | | | Alcohol consumption (“high” vs. “low”) | | | Adjustment  factor |
| --- | --- | --- | --- | --- | --- | --- | --- | --- | --- | --- | --- | --- | --- | --- | --- | --- | --- | --- | --- | --- | --- | --- | --- | --- | --- | --- |
| 1.  Katsinelos, P. 2013 | | | Northern Greece | | | | | | 2007–2010 | | | | | 1990 | | 1263/727 | | Mixed control | | | 1.78 (1.15-2.78) | | >80g/day vs. <80g /day | | | None |
| 2  Ronkainen, J. 2005 | | | Northern Sweden | | | | | | 1998 | | | | | 2860 | | 741/259 | Mixed control | | | | 3.0 (1.03-8.54) | | >50g/week vs. <50g /week | | | Adjusted for sex, age and smoking. |
| 3  Johansson, J. 2007 | | | Southeast Sweden | | | | 1997-1999 | | | | | | | 764 | | 427/92 | Endoscopy negative control | | | | 0.6 (0.2–1.7) | | user(>12.8g/day) vs. Abstainer (none) | | | Adjusted for age, gender,reflux symptoms, BMI, H. pylori and smoking |
| 4  Conio, M. 2002 | | | | Italian | | | 1995–1999 | | | | | | | 600 | | 343/112(spirits) | Endoscopy negative control | | | | 1.3 (0.8–2.0) | | Duration of spirits intake: 10 years vs. 3 years | | | Adjusted for geographic center, gender and age |
| 5  Akiyama, T. 2009 | | | | Japan | | | 2005-2006 | | | | | | | 869 | | 553/316 | Mixed control | | | | 1.31  (1.10-1.56) | | Regular drinking habit(≥12.8g/day) vs. None | | | Adjusted for age, sex, BMI, smoking, gastric mucosal atrophy and erosive esophagitis. |
| 6  Anderson, L. A. 2009 | | | Ireland | | | | 2002-2005 | | | | | | | 941 | | 151/121  151/108  151/101 | Population control | | | | 0.84 (0.44-1.57)  0.57 (0.29-1.09)  0.77 (0.40-1.51) | | >1 drink(12.8g)/month and ≤1 drinks(12.8g)/week vs. <1 drink(12.8g)/month  2-3 drinks (25.6g-38.4g)/week vs. <1 drink(12.8g)/month  >3 drinks(38.4g)/week vs. <1 drink(12.8g)/month | | | Adjusted for sex, age at interview, smoking, BMI(at age 21 years), job type, education, energy intake (kcal), fruit and vegetable intake, H pylori infection, NSAID, GERD and location |
| 7  Veugelers, P.J.  (liquor) 2006 | | | | | Canada | | | 2001-2003  2001-2003 | | | | | | 431  431 | | NA | Population control | | | | 1.20  (0.63–2.29) | | 1–40 drinks(14g-560g)/month vs. < 1 (14g)drink/month | | | Adjusted for age, gender, overweight, obesity, smoking, vitamin C and multivitamin use. |
|  |  |  |  |  |  |  |  |  |  |  |  |  |  |  |  | NA |  | | | | 3.06 (1.23–7.62) | | ≥ 40 drinks(560g)/month vs. < 1 drink(14g)/month | | |  |
| 8  Thrift. A. P. 2012 | | | Australia | | | | 2003-2006 | | | | 706 | | | | 14/9 (MEN) | | Inflammation  Control | | | | | 2.14 (0.46-9.98) | <1 standard drinks/week (<10g/week) vs. none | | | Adjusted for age, sex, smoking status, body mass index, highest level ofeducation, and frequency of use of acid suppressant medications. |
|  |  |  |  |  |  |  |  |  |  |  |  |  |  |  | 14/45 (MEN) | |  | | | | | 0.75 (0.30-1.86) | 1-6 standard drinks /week(10-60g/week) vs. none | | |  |
|  |  |  |  |  |  |  |  |  |  |  |  |  |  |  | 14/56  (MEN) | |  | | | | | 0.78 (0.32-1.92) | 7-20 standard drinks/week (70-200g/week) vs. none | | |  |
|  |  |  |  |  |  |  |  |  |  |  |  |  |  |  | 14/57  (MEN) | |  | | | | | 1.02 (0.41-6.01) | ≥21 standard drinks/week(≥210g/week) vs. none | | |  |
|  | | | | | |  |  | | | |  | | | | 23/13 (WOMEN) | | Inflammation  control | | | | | 0.59 (0.24-1.47) | <1 standard drinks/week (<10g/week) vs. none | | |  |
|  |  |  |  |  |  |  |  |  |  |  |  |  |  |  | 23/49 (WOMEN) | |  | | | | | 0.70 (0.35-1.40) | 1-6 standard drinks/week (10-60g/week) vs., none | | |  |
|  |  |  |  |  |  |  |  |  |  |  |  |  |  |  | 23/17 (WOMEN) | |  | | | | | 0.34 (0.15-0.76) | 7-20 standard drinks/week (70-200g/week) vs. none | | |  |
|  |  |  |  |  |  |  |  |  |  |  |  |  |  |  | 23/2 (WOMEN) | |  | | | | | 0.24 (0.05-1.25) | ≥21 standard drinks/week (≥210g/week) vs. none | | |  |
| 9  Steevens, J. 2011 | | | | Dutch | | | 1986–2002 | | | | 4736 | | | | 24/50  (MEN) | | Population control | | | | | 1.37  (0.81–2.31) | | >0 to <5g/day vs. abstainer | | Adjusted for age (years), cigarette smoking [current smoking status (yes/no), frequency (number of cigarettes/day), and duration (years)], BMI (kg/m2). |
|  |  |  |  |  |  |  |  |  |  |  |  |  |  |  | 24/58  (MEN) | |  | | | | | 1.28  (0.77–2.13) | | 5 to <15g/day vs. abstainer | |  |
|  |  |  |  |  |  |  |  |  |  |  |  |  |  |  | 24/52  (MEN) | |  | | | | | 1.26  (0.75-2.13) | | 15 to <30g/day vs. abstainer | |  |
|  |  |  |  |  |  |  |  |  |  |  |  |  |  |  | 24/28  (MEN) | |  | | | | | 1.02  (0.57–1.84) | | ≥30g/day vs. abstainer | |  |
|  | | | | | |  |  | | | |  | | | | 51/72  (WOMEN) | |  | | | | | 1.19  (0.82–1.73) | | >0 to <5g/day vs. abstainer | |  |
|  |  |  |  |  |  |  |  |  |  |  |  |  |  |  | 51/20  (WOMEN) | |  | | | | | 0.66  (0.38–1.16) | | 5 to <15g/day vs. abstainer | |  |
|  |  |  |  |  |  |  |  |  |  |  |  |  |  |  | 51/15  (WOMEN) | |  | | | | | 0.75  (0.40-1.41) | | 15 to <30g/day vs. abstainer | |  |
| 10  Akiyama, T.  2008 | | | Yokohama | | | | August 2005 and July 2006 | | | | 463(male) | | | | 187/80 | | Endoscopy negative control | | | | | 1.278  (0.75-2.17) | Light (< 25 g/day) vs. never drinkers | | | None |
|  |  |  |  |  |  |  |  |  |  |  |  |  |  |  | 187/87 | |  | | | | | 1.458  (0.87-2.43) | Moderate (25 – 50 g/day) vs. never drinkers | | |  |
|  |  |  |  |  |  |  |  |  |  |  |  |  |  |  | 187/109 | |  | | | | | 1.912  (1.19-3.09) | Heavy (> 50 g/day) vs. never drinkers | | |  |
| 11  Kubo, A.  2009 | | Northern California | | | | | 2002-2005 | | | | 953 | | | | 213/279 | | Inflammation  control | | | | | 1.58  (1.03-2.14) | <7 drinks/week(<91g/week) vs. no alcohol use | | | Adjusted for age, race, gender, location of diagnosis, fruit and vegetables intake, H. Pylori status, income and education |
|  |  |  |  |  |  |  |  |  |  |  |  |  |  |  | 213/100 | |  | | | | | 1.56  (0.75-3.22) | 7–13 drinks/week (91-181g /week) vs. no alcohol use | | |  |
|  |  |  |  |  |  |  |  |  |  |  |  |  |  |  | 213/44 | |  | | | | | 1.53  (0.80-2.92) | 14+ drinks/week (182g/week)vs. no alcohol use | | |  |
| 12  Yates, M.  2014 | England | | | | | | 1993 and 2008, | | | | 24068 | | | | 3094/12130 | | Population control | | | | | 0.61 (0.33–1.11) | | >0 to < 7(55.3g) units per week vs. no alcohol | | Adjusted for age and gender |
|  |  |  |  |  |  |  |  |  |  |  |  |  |  |  | 3094/4560 | |  | | | | | 0.84 (0.43–1.61) | | 7 to < 14(55.3-110.6g) units per week vs. no alcohol | |  |
|  |  |  |  |  |  |  |  |  |  |  |  |  |  |  | 3094/2014 | |  | | | | | 0.64 (0.28–1.49) | | 14 to < 21(110.6-165.9g) units per week vs. no alcohol | |  |
|  |  |  |  |  |  |  |  |  |  |  |  |  |  |  | 3094/933 | |  | | | | | 1.09 (0.45–2.61) | | 21 to <28 (165.9-221.2g) units per week vs. no alcohol | |  |
|  |  |  |  |  |  |  |  |  |  |  |  |  |  |  | 3094/1005 | |  | | | | | 0.84 (0.34–2.10) | | >28 (221.2g) units per week vs. no alcohol | |  |
| 13  Caygill, C.P.2002  (Cohort 2  :Wexham Park Case-Control Study) | | | Scotland | | | | | | | 1997.2-1997.9 | | | 338 | | 85/15  (male) | | Inflammation  control | | | | | 1.42(0.93-2.16) | >21(210g) units /week vs. <21(210g)week | | | None |
|  |  |  |  |  |  |  |  |  |  |  |  |  |  |  | 100/2  (female) | |  | | | | | 1.70(0.99-2.93) | >15 (150g) units /week vs. ≤14(140g)/week | | |  |
| 14  Thrift AP.  2011 | | | Australia | | | | 2003-2006 | | | | | | 1350 | | 33/78 MEN  (Nondysplastic BE) | | Inflammation control | | | | | 0.48 (0.19-1.26) | 1-6 standard drinks/week (10-60g/week)vs. <1 standard drinks/week(<10g/week) | | | Adjusted for age, sex, education level, cumulative  smoking history, BMI 1 year ago, frequency of heart burn or reflux symptoms in the 10-year period before study, frequency of aspirin or NSAID use, and ever use of PPIs |
|  |  |  |  |  |  |  |  |  |  |  |  |  |  |  | 33/101 MEN  (Nondysplastic BE) | |  |  |  |  |  | 0.40 (0.15-1.05) | 7-20 standard drinks/week (70-200g/week) vs. <1 standard drinks/week(<10g/week) | | |  |
|  |  |  |  |  |  |  |  |  |  |  |  |  |  |  | 33/68 MEN  (Nondysplastic BE) | |  |  |  |  |  | 0.36 (0.13-1.01) | 21-40 standard drinks/week(210-400g/week) vs. <1 standard drinks/week(<10g/week) | | |  |
|  |  |  |  |  |  |  |  |  |  |  |  |  |  |  | 33/ 20 MEN  (Nondysplastic BE) | |  |  |  |  |  | 0.44 (0.11-1.76) | ≥42 standard drinks/week(≥420g /week) vs.<1 standard drinks/week(<10g/week) | | |  |
|  |  |  |  |  |  |  |  |  |  |  |  |  |  |  | 18/58 MEN  (dysplastic BE) | | Inflammation control | | | | | 1.49 (0.32-7.01) | 1-6 standard drinks/week (10-60g/week) vs. <1 standard drinks/week(<10g/week) | | |  |
|  |  |  |  |  |  |  |  |  |  |  |  |  |  |  | 18/76  MEN  (dysplastic BE) | |  | | | | | 1.02 (0.22-4.67) | 7-20 standard drinks/week (70-200g/week) vs. <1 standard drinks/week(<10g/week) | | |  |
|  |  |  |  |  |  |  |  |  |  |  |  |  |  |  | 18/47 MEN  (dysplastic BE) | |  | | | | | 0.82 (0.17-3.98) | 21-40 standard drinks/week (210-400g/week) vs. <1 standard drinks/week (<10g/week) | | |  |
|  |  |  |  |  |  |  |  |  |  |  |  |  |  |  | 18/14 MEN  (dysplastic BE) | |  | | | | 2.80 (0.38-20.40) | | ≥42 standard drinks/week(≥420g /week) vs. <1 standard drinks/week(<10g/week) | | |  |
|  |  |  |  |  |  |  |  |  |  |  |  |  |  |  | 76/113  WOMEN  (nondysplastic BE) | | Inflammation control | | | | | 0.84 (0.44-1.61) | 1-6 standard drinks/week(10-60g/week) vs. <1 standard drinks/week  (<10g/week) | | |  |
|  |  |  |  |  |  |  |  |  |  |  |  |  |  |  | 76/66 WOMEN  (nondysplastic BE) | |  | | | | | 0.46 (0.20-1.07) | ≥7 standard drinks/week (≥70g/week) vs. <1 standard drinks/week(<10g/week) | | |  |
|  |  |  |  |  |  |  |  |  |  |  |  |  |  |  | 49/77 WOMEN  (dysplastic BE) | | Inflammation control | | | | | 0.48（0.10-2.26） | | | 1-6 standard drinks/week (10-60g/week) vs. <1 standard drinks/week (<10g/week) |  |
|  |  |  |  |  |  |  |  |  |  |  |  |  |  |  | 49/51 WOMEN  (dysplastic BE) | |  | | | | | 0.01（0.00-0.29） | | | ≥7 standard drinks/week (≥70g/week) vs. <1 standard drinks/week (<10g/week) |  |
| 15  Thrift, A. P.  2014 | | Houston VA | | | | | 2008-2012 | | | | 1856 | | | | 134/357 | | Endoscopy negative control | | | | | 1.01  (0.57-1.78) | <7 (70g) standard drinks/week vs. non-drinker | | | Adjusted for age, sex, race, duration of GERD symptoms, WHR, H. pylori infection, PPI use and NSAID use |
|  |  |  |  |  |  |  |  |  |  |  |  |  |  |  | 134/190 | |  | | | | 1.27  (0.68–2.36) | | 7 to<14 standard drinks/week vs. .non-drinker  (≥70g/week) | | |  |
|  |  |  |  |  |  |  |  |  |  |  |  |  |  |  | 134/377 | |  | | | | 0.84 (0.44–1.63) | | 14 to<28 standard drinks/week vs. non-drinker (≥70g/week) | | |  |
|  |  |  |  |  |  |  |  |  |  |  |  |  |  |  | 113/79 | |  | | | | 1.11 (0.63–1.95) | | ≥28 standard drinks/week vs. non-drinker (≥280g/week) | | |  |

NA, not applicable.

BMI: body mass index

NSAID: non steroidal anti inflammatory drug

GERD: gastroesophageal reflux disease

PPI: proton-pump inhibitor

WHR: waist hip rate
